# Parvalbumin^+^ interneurons obey unique connectivity rules and establish a powerful lateral-inhibition microcircuit in dentate gyrus

**DOI:** 10.1038/s41467-018-06899-3

**Published:** 2018-11-02

**Authors:** Claudia Espinoza, Segundo Jose Guzman, Xiaomin Zhang, Peter Jonas

**Affiliations:** 1 IST Austria (Institute of Science and Technology Austria), Am Campus 1, 3400 Klosterneuburg, Austria; 2 Present Address: Institute for Molecular Biotechnology (IMBA), Dr. Bohr-Gasse 3, 1030 Wien, Austria

## Abstract

Parvalbumin-positive (PV^+^) GABAergic interneurons in hippocampal microcircuits are thought to play a key role in several higher network functions, such as feedforward and feedback inhibition, network oscillations, and pattern separation. Fast lateral inhibition mediated by GABAergic interneurons may implement a winner-takes-all mechanism in the hippocampal input layer. However, it is not clear whether the functional connectivity rules of granule cells (GCs) and interneurons in the dentate gyrus are consistent with such a mechanism. Using simultaneous patch-clamp recordings from up to seven GCs and up to four PV^+^ interneurons in the dentate gyrus, we find that connectivity is structured in space, synapse-specific, and enriched in specific disynaptic motifs. In contrast to the neocortex, lateral inhibition in the dentate gyrus (in which a GC inhibits neighboring GCs via a PV^+^ interneuron) is ~ 10-times more abundant than recurrent inhibition (in which a GC inhibits itself). Thus, unique connectivity rules may enable the dentate gyrus to perform specific higher-order computations.

## Abstract (web summary)

GABAergic interneurons are known to provide inhibition to allow computational function of neuronal network. Here, Espinoza and colleagues show that connectivity of granule cells and interneurons in the dentate gyrus of mouse hippocampus are consistent with the circuit architecture capable of performing a winners-take-all mechanism.

## Introduction

Throughout the brain, fast-spiking, parvalbumin-expressing (PV^+^) GABAergic interneurons play a key role in several higher functions, such as feedforward and feedback inhibition, high-frequency network oscillations, and pattern separation^1^. Understanding how PV^+^ interneurons contribute to these complex computations requires a detailed and quantitative analysis of their synaptic connectivity. While early studies suggested that connectivity of PV^+^ interneurons is random^2^, more recent work highlighted several specific connectivity rules^3–7^ (Supplementary Table 1). Analysis of principal neuron (PN)–interneuron (IN) connectivity in the neocortex revealed that reciprocally connected pairs occurred much more frequently than expected in a random network^3–7^. Moreover, synaptic strength appeared to be higher in these reciprocally connected motifs^4,6^. Whether these connectivity rules also apply in other microcircuits, such as the hippocampus, has not been determined yet.

Pattern separation is a fundamental network computation in which PV^+^ interneurons are likely to be involved. Pattern separation is thought to be particularly important in the dentate gyrus, where conversion of overlapping synaptic input patterns into non-overlapping action potential (AP) output patterns^8–12^ may facilitate reliable storage of information in the downstream CA3 network^9,13,14^. Previous studies suggested a model of pattern separation based on a winner-takes-all mechanism mediated by feedback inhibition^15–19^. Such a model has received experimental support in the olfactory system^20–22^. While some studies suggested that similar mechanisms may operate in the dentate gyrus^23,24^, it is not clear whether the rules of PN–IN connectivity are adequate to support such a model. Specifically, two forms of feedback inhibition need to be distinguished: recurrent inhibition, in which an active PN inhibits itself via reciprocal PN–IN connections, and lateral inhibition, in which an active PN inhibits neighboring PNs but not itself^25,26^. A winner-takes-all mechanism likely requires lateral inhibition; recurrent inhibition may be counter-productive, because it could suppress potential winners^17,26,27^. However, in both neocortex and brain areas directly connected to the hippocampus, recurrent inhibition and lateral inhibition are equally abundant^3–7^ (Supplementary Table 1). Such a circuit design would seem incompatible with efficient pattern separation.

To resolve this apparent contradiction, we examined the functional connectivity rules in PN–IN networks in the dentate gyrus, using simultaneous recordings from up to seven granule cells (GCs) and up to four GABAergic interneurons. Our experiments reveal a uniquely high abundance of lateral inhibition mediated by PV^+^ interneurons.

## Results

### Octuple recordings from neurons in the dentate gyrus

**Fig. 1:**
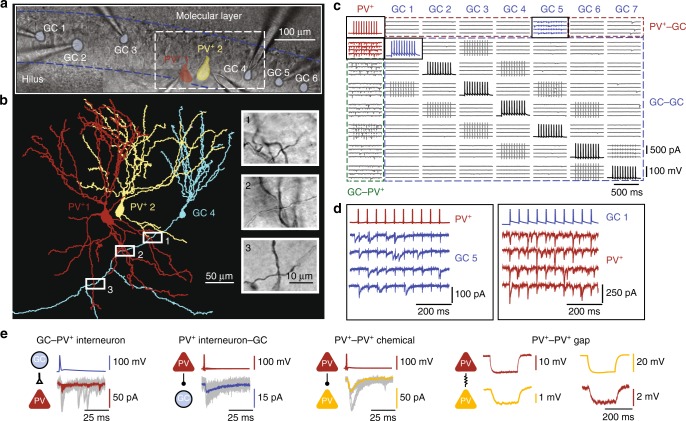
Octuple recording from GCs and PV^+^ interneurons in the dentate gyrus. **a** Octuple recording from five GCs and two PV^+^ interneurons (seven cells successfully recorded). Infrared differential interference contrast video micrograph of the dentate gyrus in a 300-µm slice preparation, with eight recording pipettes. Shaded areas represent the 2D projections of cell bodies (blue, GCs; red and yellow, PV^+^ interneurons). Blue dashed lines, boundaries of GC layer. **b** Partial reconstruction of one GC and two PV^+^ interneurons in the same recording as shown in (**a**). Cells were filled with biocytin during recording and visualized using 3,3′-diaminobenzidine as chromogen. For clarity, only the somatodendritic domains were drawn for the PV^+^ interneurons. Insets, biocytin-labeled putative synaptic contacts, corresponding to boxes in main figure. **c** Connectivity matrix of an octuple recording (all eight cells successfully recorded). Subpanels on the diagonal (AP traces) represent the presynaptic cells, subpanels outside the diagonal (EPSC or IPSC traces) indicate postsynaptic cells. In this example, 56 connections were tested; 7 excitatory GC–PV^+^ interneuron connections, 7 inhibitory PV^+^ interneuron–GC connections, and 42 connections between GCs. Brief transients in a subset of traces represent capacitive coupling artifacts, as shown in previous publications^5, 14^. **d** Expanded view of presynaptic APs and postsynaptic currents, corresponding to the boxed areas in (**c**). In this octuple recording, an inhibitory synaptic connection was identified between the PV^+^ interneuron (red) and GC 5 (blue) and an excitatory synaptic connection was found between GC 1 (blue) and the PV^+^ interneuron (red). The presence of a unidirectional excitatory GC–PV^+^ interneuron connection and a unidirectional inhibitory PV^+^ interneuron–GC connection documents the existence of lateral inhibition in this recording. **e** Coexistence of different synapses in an octuple recording. In this recording, an excitatory GC–PV^+^ interneuron connection, an inhibitory PV^+^ interneuron–GC connection, a chemical inhibitory connection between the PV^+^ interneurons, and an electrical connection between the PV^+^ interneurons were found (from left to right). Same recording as in (**a**) and (**b**)

To determine the functional connectivity rules between PNs and INs in the dentate gyrus, we performed simultaneous whole-cell recordings from up to eight neurons (up to seven GCs and up to four INs) in vitro (Fig. 1a, b). PV^+^ interneurons, somatostatin-positive (SST^+^), and cholecystokinin-positive (CCK^+^) interneurons were identified in genetically modified mice, obtained by crossing Cre or Flp recombinase-expressing lines with tdTomato or EGFP reporter lines. PV^+^ interneurons showed the characteristic fast-spiking AP phenotype during sustained current injection, whereas both SST^+^ and CCK^+^ interneurons generated APs with lower frequency, corroborating the reliability of the genetic labeling (Supplementary Figure 1).

**Fig. 2:**
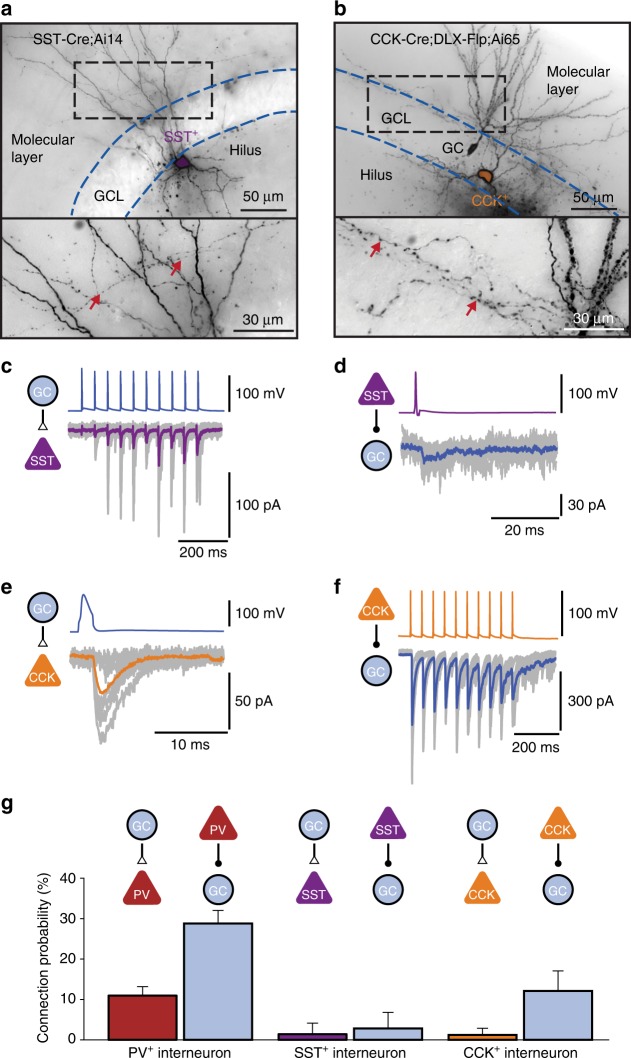
Differential connectivity of PV^+^, CCK^+^, and SST^+^ interneurons in the dentate gyrus. **a** Light micrograph of a SST^+^ interneuron filled with biocytin during recording, and visualized using 3,3′-diaminobenzidine as chromogen. Cells were identified by genetic labeling in SST-Cre mice. Axon branches in the molecular layer (red arrows) suggest that the cell was a HIPP or TML interneuron^68, 69^. GCL, granule cell layer. **b** Light micrograph of a CCK^+^ interneuron filled with biocytin. Cells were identified by genetic labeling in CCK-Cre;DLX 5/6-Flp mice. Axon branches in the inner molecular layer (red arrows) suggest that the cell was a HICAP interneuron^68–70^. **c**, **d** Excitatory and inhibitory connectivity of SST^+^ interneurons. GC–SST^+^ interneuron unitary EPSCs are shown in (**c**), SST^+^ interneuron–GC IPSCs are illustrated in (**d**). Individual synaptic responses (gray) and average trace (magenta or blue, 15 traces) are shown overlaid. Note the facilitation of EPSCs during train stimulation in (**c**). **e**, **f** Excitatory and inhibitory connectivity of CCK^+^ interneurons. GC–CCK^+^ interneuron EPSCs are shown in (**e**), CCK^+^ interneuron–GC IPSCs are illustrated in (**f**). Note the asynchronous release during and after train stimulation in (**f**), which is highly characteristic of CCK^+^ interneuron output synapses^70^. **g** Comparison of average connection probability for pairs with an intersomatic distance of ≤ 100 µm. Whereas PV^+^ interneurons were highly connected, SST^+^ and CCK^+^ interneurons showed a markedly lower excitatory and inhibitory connectivity (number of tested connections 767, 71, and 165). Error bars represent 95%-confidence intervals estimated from a binomial distribution

To probe synaptic connectivity, we stimulated presynaptic neurons under current-clamp conditions, and recorded excitatory postsynaptic currents (EPSCs) or inhibitory postsynaptic currents (IPSCs) in postsynaptic neurons in the voltage-clamp configuration (Fig. 1c–e, Fig. 2). In total, we tested 9098 possible connections in 50 octuples, 72 septuples, 68 sextuples, 48 quintuples, 17 quadruples, 10 triples, and 5 pairs in 270 slices. Interestingly, PV^+^ interneurons showed a much higher connectivity than both SST^+^ and CCK^+^ interneurons. For GC–PV^+^ interneuron pairs with intersomatic distance ≤ 100 µm, the mean connection probability was 11.0% for excitatory GC–PV^+^ interneuron and 28.8% for inhibitory PV^+^ interneuron–GC connectivity (Fig. 2g). In contrast, for both SST^+^ interneurons and CCK^+^ interneurons, the mean connection probability was substantially lower (1.4 and 2.8% for SST^+^ interneurons, 1.2 and 12.1% for CCK^+^ interneurons; Fig. 2g). Excitatory interactions between GCs were completely absent, and disynaptic inhibitory interactions between GCs^28,29^ were extremely sparse (0.124%). These results indicate that in the dentate gyrus PV^+^ interneurons show a markedly higher connectivity than SST^+^ and CCK^+^ interneurons, extending previous observations in the neocortex^30^.

### Connectivity rules for excitatory input of PV^+^ interneurons

**Fig. 3:**
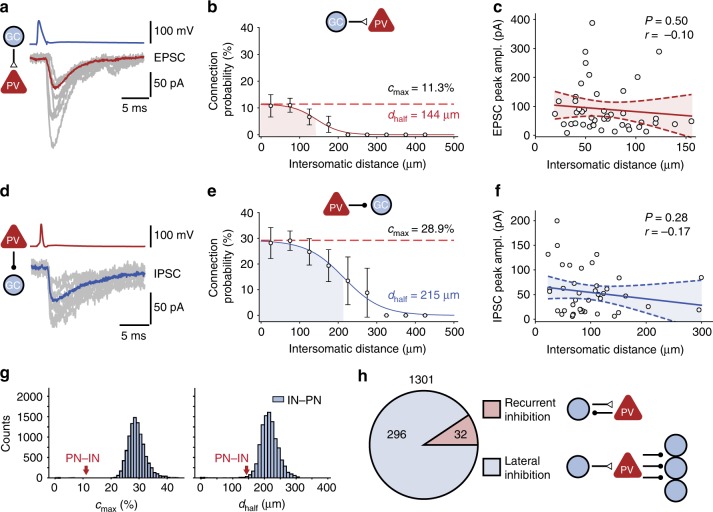
Rules of excitatory and inhibitory connectivity in GC–PV^+^ interneuron networks. **a** Unitary EPSCs, with individual synaptic responses (gray) and average trace (red, 15 traces) in a representative GC–PV^+^ interneuron pair. **b** GC–PV^+^ interneuron connection probability plotted versus intersomatic distance. Connection probability was determined as the ratio of the number of found connections over that of all possible connections in a given distance range. Error bars represent 95%-confidence intervals estimated from a binomial distribution. Data points were fit with a sigmoidal function; shaded area indicates the distance range in which connection probability decayed to half-maximal value (space constant). Red dashed line, maximal connection probability. Maximal connection probability (*c*_max_) was 11.3%, and space constant (*d*_half_) was 144 µm. **c** Peak amplitude of unitary EPSCs at GC–PV^+^ interneuron synapses, plotted against intersomatic distance. Data points were fit by linear regression; dashed lines indicate 95%-confidence intervals. **d**–**f** Similar plots as shown in (**a**–**c**), but for IPSCs generated at inhibitory PV^+^ interneuron–GC synapses. Maximal connection probability was 28.9%, and space constant was 215 µm. **g** Bootstrap analysis of maximal connection probability and space constant. Histograms indicate distributions of *c*_max_ (left) and *d*_half_ (right) for 10,000 bootstrap replications of the inhibitory PV^+^ interneuron–GC connections. Red arrows indicate experimental mean values for GC–PV^+^ interneuron synapses. **h** Number of reciprocally coupled GC–PV^+^ interneuron pairs (excitatory and inhibitory synapse; “recurrent inhibition motif”) and unidirectionally coupled PV^+^ interneuron–GC pairs (inhibitory synapse only; “lateral inhibition motif”). Note that the number of lateral inhibition motifs was almost 10-times higher than that of recurrent inhibition motifs, demonstrating the high abundance of lateral inhibition in the dentate gyrus microcircuit

As PV^+^ interneurons showed the highest input and output connectivity, we focused our functional connectivity analysis on this interneuron subtype. We first examined the rules of excitatory synaptic connectivity between GCs and PV^+^ interneurons by measuring EPSCs (Fig. 3a–c). We found that PV^+^ interneurons were highly and locally connected to GCs. The connection probability showed a peak of 11.3%, and steeply declined as a function of intersomatic distance, with a space constant of 144 µm (Fig. 3b). In contrast, the EPSC peak amplitude showed no significant distance dependence (Fig. 3c). To determine the efficacy of unitary GC–PV^+^ interneuron connections, we measured unitary excitatory postsynaptic potentials (EPSPs). Unitary EPSPs had a mean peak amplitude of 1.79 ± 0.36 mV (range: 0.30–7.16 mV; Supplementary Figure 2a, b)^28,31,32^. To assess the efficacy of these events in triggering spikes in the presence of ongoing synaptic activity from multiple sources, we performed in vivo whole-cell recordings from fast-spiking interneurons in the dentate gyrus in awake mice running on a linear treadmill (Supplementary Figure 2c–g). Under in vivo conditions, the difference between baseline membrane potential and threshold was 10.3 ± 1.8 mV (three in vivo recordings from fast-spiking interneurons in dentate gyrus). Thus, although the largest unitary EPSPs were close to the threshold of AP initiation, they were insufficient to trigger a spike. However, the high focal GC–PV^+^ interneuron connectivity (Fig. 3b) may enable activation of PV^+^ interneurons by spatial summation.

### Connectivity rules for inhibitory output of PV^+^ interneurons

Next, we examined the rules of inhibitory synaptic connectivity between GCs and PV^+^ interneurons by measuring IPSCs (Fig. 3d–f). Similar to excitatory GC–PV^+^ interneuron connectivity, inhibitory PV^+^ interneuron–GC connectivity was distance-dependent (Fig. 3e). However, maximal connection probability was higher (28.9%) and the range of connectivity was wider (215 µm) than that of excitation. Bootstrap analysis revealed that both maximal connectivity and space constant were significantly shorter for excitatory GC–PV^+^ interneuron synapses than for inhibitory PV^+^ interneuron–GC synapses (*P* < 0.0001 and *P* = 0.0042, respectively; Fig. 3g). Thus, different connectivity rules apply for excitatory and inhibitory GC –PV^+^ interneuron connections (focal excitation versus broad inhibition).

To compare the connectivity rules in the dentate gyrus with those in other brain regions, we quantified the ratio of excitatory to inhibitory connection probability. We found that inhibition was much more abundant than excitation, with a connection probability ratio of 3.83, substantially higher than in other brain areas (Supplementary Table 1). Furthermore, we quantified the abundance of lateral and recurrent motifs in pairs of neurons. In our total sample of 1301 GC–PV^+^ interneuron pairs, we found 296 unidirectional inhibitory connections, but only 32 bidirectional connections (Fig. 3h). Thus, the ratio of lateral inhibition to recurrent inhibition was 9.25, substantially higher than in other circuits (Supplementary Table 1). These results indicate that connectivity rules of PV^+^ interneurons in the dentate gyrus are unique in comparison to other previously examined circuits.

### Connectivity rules for mutual inhibition of PV^+^ interneurons

**Fig. 4:**
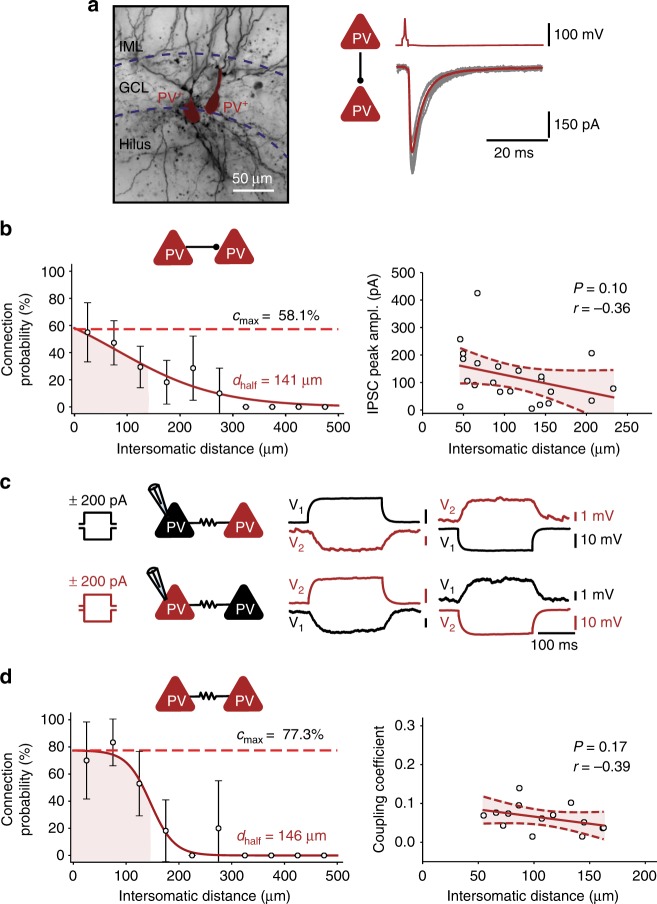
Rules of chemical and electrical connectivity between PV^+^ interneurons. **a** Left, light micrograph of a biocytin-labeled PV^+^ interneuron–PV^+^ interneuron pair. Right, unitary IPSCs, with individual synaptic responses (gray) and average trace (red, 15 traces) in the same pair. GCL, granule cell layer; IML, inner molecular layer. **b** PV^+^ interneuron–PV^+^ interneuron chemical connection probability (left) and IPSC peak amplitude (right) plotted versus intersomatic distance. Connection probability data points were fit with a sigmoidal function, IPSC amplitude data were analyzed by linear regression. Maximal connection probability was 58.1%, and space constant was 141 μm. **c** Electrical coupling between two PV^+^ interneurons. Voltage changes in the pre- and postsynaptic cell caused by the injections of long polarizing current pulses (left, + 200 pA; right, −200 pA; 200 ms) in one of the coupled cells. **d** PV^+^ interneuron–PV^+^ interneuron electrical connection probability (left) and coupling coefficient (right) plotted versus intersomatic distance. Maximal connection probability was 77.3%, and space constant was 146 μm. The coupling coefficient (CC) was calculated as the mean ratio of steady-state voltages (*V*_2_/*V*_1_, *V*_1_/*V*_2_) during application of current pulses in one of the cells (cell 1 and cell 2, respectively)

Finally, we analyzed the functional connectivity rules for synapses between interneurons (Fig. 4). Chemical inhibitory synapses between PV^+^ interneurons showed a connectivity pattern that was more focal than that of inhibitory PV^+^ interneuron–GC synapses (Fig. 4a, b). Likewise, electrical synapses between PV^+^ interneurons^33–35^ showed a focal connectivity pattern (Fig. 4c, d). Bootstrap analysis revealed that the maximal connectivity was significantly higher, while the space constant was significantly shorter for inhibitory PV^+^–PV^+^ interneuron synapses than for PV^+^ interneuron–GC synapses (*P* = 0.0001 and *P* = 0.0036, respectively). Furthermore, recordings from GCs and multiple PV^+^ interneurons provided direct evidence for the suggestion^33^ that EPSPs propagate through gap junctions, although the peak amplitude is markedly attenuated (Supplementary Figure 3). Taken together, these results indicate that connectivity rules in PN–IN microcircuits are synapse-specific. Different connectivity rules apply to excitatory and inhibitory synapses between PNs and INs (GC–PV^+^ versus PV^+^–GC), and to inhibitory synapses terminating on different postsynaptic target cells (PV^+^–GC versus PV^+^–PV^+^ synapses).

### Disynaptic connectivity motifs

**Fig. 5:**
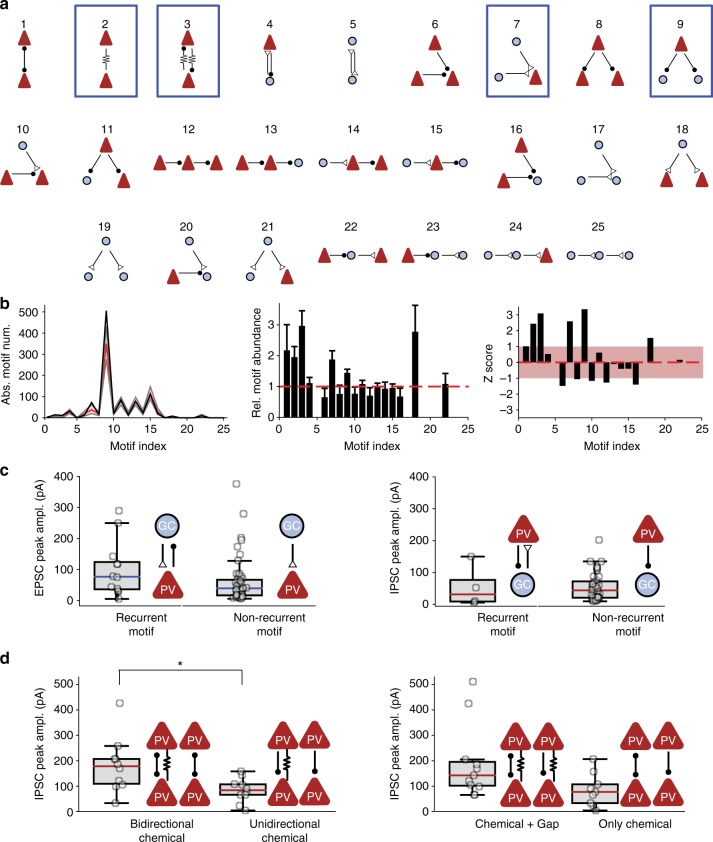
Overabundance of disynaptic connectivity motifs in GC–PV^+^ interneuron networks and different functional properties of synapses embedded in motifs. **a** Graph analysis of disynaptic connectivity motifs. In total, there are five possible disynaptic connectivity motifs with two cells and 20 disynaptic motifs involving three cells. Arrows with open triangles indicate excitatory synapses, arrows with filled circles represent inhibitory synapses, and arrows with zigzag lines indicate gap junctions. Number indicates motif index. **b** Analysis of the number of motifs in 10,000 simulated data sets. Connection probability for the simulated data set was specified according to the experimentally determined spatial rules. Left, absolute motif number in experimental (black) and simulated data set (red, median; gray, 90%-confidence interval). Center, bar plot of relative abundance of various motifs (number of motifs in experimental data set over mean number in simulated data set). Error bars were taken from bootstrap analysis. Right, bar plot of z score of the different motifs. Light red area indicates z score in the interval [−1, 1]. Motifs 2, 3, 7, and 9 were significantly enriched above the chance level (*P* = 0.03145, 0.0085, 0.0272, and 0.0068 after multiple comparison correction). In contrast, motifs 6, 8, 10, 12, and 16 were slightly, but not significantly underrepresented (*P* = 0.15 for motif 6). Note that motifs 5, 17, 19–21, and 23–25 were not encountered in the present data set, because of the lack of connectivity between GCs. **c** Comparison of EPSC peak amplitude (left) and IPSC peak amplitude (right) in bidirectionally versus unidirectionally coupled GC–PV^+^ interneuron pairs. Peak amplitudes were not significantly different (*P* = 0.33 and 0.58, respectively). **d** Comparison of IPSC peak amplitude in PV^+^ interneuron–PV^+^ interneuron pairs connected by different chemical or electrical synapse motifs. IPSC peak amplitude was significantly larger in pairs with bidirectional inhibitory connections than with unidirectional connections (*P* = 0.016) and slightly higher in connections with than without gap junctions (*P* = 0.057). Asterisk indicates *P* < 0.05

Previous studies demonstrated that recurrent PN–PV^+^ interneuron connectivity motifs are enriched above the chance level expected for a random network in several cortical microcircuits^3–7,36^. To test this hypothesis, we analyzed the abundance of all 25 possible disynaptic connectivity motifs in our sample (Fig. 5)^37^. To probe whether connectivity was random^38^ or nonrandom^14,39–42^, we compared motif numbers in our experimental data to a simulated data set assuming random connectivity with experimentally determined distance-dependent connection probabilities (Fig. 5a, b).

Among the 25 possible disynaptic motifs, four types of motifs were significantly enriched above the chance level: (1) Gap junction connections between PV^+^ interneurons, (2) mutual inhibition motifs (PV^+^ interneuron–PV^+^ interneuron connections) combined with gap junction connections^43^, (3) convergence motifs (connections of multiple GCs on a single PV^+^ interneuron), and (4) divergence motifs (connections of one PV^+^ interneuron onto multiple GCs; Fig. 5b; *P* < 0.05 after correction for multiple comparisons). Surprisingly, reciprocal GC–PV^+^ interneuron motifs were not significantly enriched.

Previous studies further demonstrated that the amplitude of unitary IPSCs is higher in bidirectionally than in unidirectionally connected PN–IN pairs^4,6^. In contrast, in the dentate gyrus neither the amplitude of EPSCs nor that of IPSCs was significantly different between bidirectionally and unidirectionally connected GC–PV^+^ interneuron pairs (Fig. 5c). However, the amplitude of IPSCs was significantly larger in PV^+^ interneuron–PV^+^ interneuron pairs coupled by reciprocal inhibitory synapses (Fig. 5d). Taken together, these results indicate that in the dentate gyrus, like in other cortical areas, synaptic connectivity of PV^+^ interneurons is nonrandom. However, both the types of enriched motifs and the rules setting synaptic strength differ from those in other circuits^3,4^.

## Discussion

Our results demonstrate that the rules of functional connectivity in the PN–IN network of the dentate gyrus fundamentally differ from those in other cortical circuits. In the dentate gyrus, unidirectionally inhibitory connections are ~10-times more frequent than reciprocal connections, demonstrating a massive prevalence of lateral inhibition in this circuit (Supplementary Table 1). In contrast, in neocortex, entorhinal cortex, and presubiculum, reciprocal connections are equally or more abundant than unidirectional connections, implying powerful recurrent inhibition^3–7^ (Supplementary Table 1). Furthermore, in the dentate gyrus mutual inhibition motifs, convergence motifs, and divergence motifs are statistically overrepresented. In contrast, in the neocortex, interneuron connectivity has been suggested to be largely random^2^. Collectively, these results suggest that the dentate gyrus network obeys unique connectivity rules.

The specific connectivity rules of the dentate circuit raise the intriguing possibility that these rules represent an adaptation to specific network functions implemented in this brain region. A major function of the dentate gyrus is pattern separation^8–12^, thought to be generated by a “winner-takes-all” mechanism^15–19^. In an ideal pattern separation circuit, a small population of activated “winner cells” must be able to efficiently and rapidly inhibit a large population of “non-winner cells”. The dentate gyrus connectivity rules are well suited for these functions. First, powerful lateral inhibition efficiently suppresses non-winners, whereas winners remain unaffected. Second, the combination of local connectivity and rapid axonal signaling mechanisms of PV^+^ interneurons^1,44^ implements a high-speed suppression mechanism, as required for efficient pattern separation. Previous modeling work suggested that scale-free network organization and the presence of hub neurons may enhance the robustness of network computations^45,46^. Our results may support this view, since the high abundance convergence and divergence motifs are consistent with scale-free architectural properties.

Furthermore, the connectivity rules of the PN–IN network may be important for the generation of network oscillations in dentate gyrus^47^. In particular, the high chemical and electrical IN–IN connectivity establishes an efficient gamma oscillator circuit. The dense and focal electrical–chemical connectivity may explain the high power and frequency of gamma oscillations in the dentate gyrus^47–49^. Previous modeling work suggested that the small-world interneuron network architecture will support the emergence of coherent gamma oscillations^50,51^. Our results support this notion, since the high abundance of electrical–chemical IN–IN motifs would be consistent with small-world architectural properties^52^. The establishment of a robust gamma oscillation circuit may, conversely, be important for the pattern separation process. Proposed models of pattern separation imply that the separation of patterns takes place in the time period during the recovery from a preceding gamma cycle^17^. Whether and how the pattern separation computation and the generation of gamma oscillations can coexist in the same circuit remains to be determined.

Our results suggest the possibility that the uniquely high abundance of lateral inhibition in dentate gyrus may contribute to pattern separation (Supplementary Table 1). What then is the function of recurrent inhibition in all other brain areas, such as the neocortical circuits? In the neocortex, PN activity is high, which requires a mechanism to establish excitation–inhibition balance; reciprocal PN–IN connectivity seems well suited for this purpose^7,20^. In contrast, in the dentate gyrus PN activity is low, and such a balancing function may not be required^53–57^. Additionally, reciprocal PN–IN connectivity could contribute to the generation of slower network oscillations in these brain regions, for example in the lower gamma or beta frequency range, which are characteristic for the neocortex.

Our results are consistent with the idea that local connectivity rules can shape diverse network computations across multiple circuits. In the dentate gyrus, the unique PN–IN connectivity rules may determine the properties of pattern separation, grid-to-place code conversion, or processing of context information^17,58^. In the neocortex, PN–IN connectivity may determine network stability and excitation–inhibition balance^7,20^. In the hippocampal CA3 network, functional PN–PN connectivity rules shape pattern completion^14^, whereas in the neocortex functional PN–PN connectivity may shape response properties such as orientation selectivity^41^. Thus, the present results contribute to the emerging view that local connectivity rules are major determinants of higher computations in neuronal networks. Future work will be needed to test this hypothesis in both network models and behavioral experiments.

## Methods

### Hippocampal slice preparation

Experiments on genetically modified mice were performed in strict accordance with institutional, national, and European guidelines for animal experimentation and were approved by the Bundesministerium für Wissenschaft, Forschung und Wirtschaft of Austria (A. Haslinger, Vienna; BMWFW-66.018/0007-WF/II/3b/2014; BMWF-66.018/0010-WF/V/3b/2015; BMWFW-66.018/0020-WF/V/3b/2016).

To genetically label PV^+^ interneurons, C57BL/6 J PV-Cre knockin mice (http://jaxmice.jax.org/strain/008069) crossed with Ai14 loxP-flanked red fluorescent protein tdTomato reporter mice (https://www.jax.org/strain/007914) were used. To identify SST^+^ interneurons, somatostatin-ires-Cre mice (C-SSTtm1Npa, kindly provided by H. van der Putten; Novartis Pharma; MTD37295, Basel, Switzerland) were crossed with Ai14 tdTomato reporter mice. Finally, to label CCK^+^ interneurons, CCK-ires-Cre;DLX 5/6-Flp mice (https://www.jax.org/strain/012706 and https://www.jax.org/strain/010815) were crossed with dual reporter mice expressing either EGFP or tdTomato (RCE = R26R CAG boosted EGFP mice, https://www.jax.org/strain/010812; Ai65, https://www.jax.org/strain/021875)^59^. Mice (20- to 44-days-old; mostly postnatal day 20–25) of either sex were lightly anesthetized with isoflurane (Forane, AbbVie, Vienna). For animals up to postnatal day 30, mice were sacrificed by decapitation. For animals older than 30 days, transcardial perfusion was performed with ice-cold sucrose-artificial cerebrospinal fluid (sucrose-ACSF) solution. Animals were deeply anesthetized with isoflurane followed by the intraperitoneal injection of a mixture of xylazine (0.5 ml, 2%), ketamine (1 ml, 10%), acepromazine (0.3 ml, 1.4%), and physiological NaCl solution (1.5 ml, 0.9%). Anesthetics were applied at a dose of 0.033 ml/10 g body weight. The depth of the anesthesia was verified by the absence of toe pinch reflexes.

For preparing slices, the brain was rapidly removed and immersed in ice-cold sucrose-ACSF solution during dissection. A block of tissue containing the hippocampus was transferred to a vibratome (VT 1200, Leica) and transverse slices of 300-µm thickness were cut with blade oscillation amplitude of 1.25 mm and blade forward movement velocity of 0.03 mm s^−1^^60^. Finally, slices were incubated at ~35 °C in standard artificial cerebrospinal fluid (ACSF) for 30 minutes and subsequently maintained at ~22 °C for maximally 5 h before transfer into the recording chamber.

### Solutions and chemicals

The ACSF used for in vitro recordings contained 125 mM NaCl, 25 mM NaHCO_3_, 25 mM glucose, 2.5 mM KCl, 1.25 mM NaH_2_PO_4_, 2 mM CaCl_2_, and 1 mM MgCl_2_. The sucrose-ACSF used for dissection contained 64 mM NaCl, 25 mM NaHCO_3_, 10 mM glucose, 120 mM sucrose, 2.5 mM KCl, 1.25 mM NaH_2_PO_4_, 0.5 mM CaCl_2_, and 7 mM MgCl_2_. The osmolarity of the solutions was 290–315 mOsm and the pH was maintained at ~7.3 when equilibrated with a 95% O_2_/5% CO_2_ gas mixture. The intracelluar solution for in vitro recordings contained 120 mM K-gluconate, 40 mM KCl, 2 mM MgCl_2_, 2 mM Na_2_ATP, 10 mM HEPES, 0.1 mM EGTA, and 0.3% biocytin, pH adjusted to 7.28 with KOH. Chemicals were purchased from Merck or Sigma-Aldrich.

### Multi-cell recordings

Glass micropipettes were fabricated from thick-walled borosilicate tubing (2 mm outer diameter, 1 mm inner diameter) and had open-tip resistances of 3–8 MΩ. They were manually positioned with eight LN mini 25 micromanipulators (Luigs and Neumann) under visual control^14^ provided by a modified Olympus BX51 microscope equipped with a 60x water-immersion objective (LUMPlan FI/IR, NA = 0.90, Olympus, 2.05  mm working distance) and infrared differential interference contrast video microscopy and epifluorescence. To preserve connectivity, cell bodies ~30–120 μm below the surface of the slice were targeted for recording. Interneurons were identified on the basis of tdTomato or EGFP fluorescence in epifluorescence illumination and the AP phenotype upon 1-s current pulses (>50 Hz in a series of pulses of 100–1,200 pA for PV^+^ interneurons). Mature GCs were identified on the basis of morphological appearance in the infrared image and on the basis of passive and active membrane properties. Cells with input resistance > 500 MΩ, potentially representing newborn GCs^61^, were not included in the analysis. Cells with resting potentials more positive than −55 mV were immediately discarded. In total, the number of successfully recorded cells per recording varied between eight and two. Recording temperature was ~22 °C (range: 20–24 °C, room temperature).

Electrical signals were acquired with four two-channel Multiclamp 700B amplifiers (Molecular Devices), low-pass filtered at 6–10 kHz, and digitized at 20 kHz with a Cambridge Electronic Design 1401 mkII AD/DA converter using custom-made stimulation-acquisition scripts running under Signal 6.0 software (CED). For current-clamp recordings, pipette capacitance was ~80% compensated and series resistance was balanced by the bridge circuit of the amplifier; settings were readjusted throughout the experiment when necessary. For voltage-clamp recordings, series resistance was not compensated, but repeatedly monitored using 2-mV hyperpolarizing pulses.

To test for synaptic connections, a presynaptic neuron was stimulated with a train of five or ten current pulses (2 ms, 1–2 nA) at frequencies of 20 or 50 Hz, while all other neurons were voltage-clamped at −70 mV (Fig. 1c). A connection was defined as monosynaptic if synaptic currents had latencies ≤ 4.0 ms and peak amplitudes were larger than 2.5 times the standard deviation of the baseline of the average trace (computed from 15–30 individual traces). Events with latencies > 4.0 ms were considered polysynaptic. For distal SST^+^–GC synapses, connectivity may be underestimated, because of  substantial attenuation of synaptic signals by cable filtering. 

### Data analysis

Recordings were analyzed using Stimfit and Python-based scripts^62^. Synaptic latency was measured from the peak of the presynaptic AP to the onset of the postsynaptic potential or current. Kinetic analysis of EPSCs or IPSCs was performed in pairs with series resistance of < 15 MΩ. Distance was measured from soma center to soma center. Analysis of the axonal arbor of PV^+^ interneurons and GCs revealed that the axonal length was 2.21 ± 0.20 and 1.59 ± 0.07 times larger than the corresponding intersomatic distance (Supplementary Figure 4). Connection probability was calculated as number of connected pairs over total number of tested pairs in each 50-µm distance interval. 95%-confidence intervals were obtained according to binomial distributions. Distance dependence of connectivity was fit with a sigmoidal function *f*(*x*) = *A* [1 + Exp[(*x* – *B*)/*C*]^−1^, where *x* is absolute distance, and *A*, *B*, and *C* are fitted parameters. Throughout the text, the maximal connection probability (*c*_max_) was determined as *f*(0), and the space constant (*d*_half_) was determined as the x’ value that specified the condition *f*(*x*’)/*f*(0) = 0.5. To test whether connectivity differed between synapses, 10,000 bootstrap replications of the inhibitory PV^+^ interneuron–GC data set were obtained, and the mean values of the GC–PV^+^ interneuron and PV^+^ interneuron–PV^+^ interneuron experimental data sets were compared against the simulated distribution^63^. Values are given as mean ± standard error of the mean (S.E.M.). Box plots show lower quartile (Q1), median (horizontal line), and upper quartile (Q3). The interquartile range (IQR = Q3–Q1) is represented as the height of the box. Whiskers extend to the most extreme data point that is no more than 1.5 x IQR from the edge of the box (Tukey style). Statistical comparisons were done either with a non-parametric Mann–Whitney *U* two-sided test or by linear regression, testing whether the slope was significantly different from 0.

To test whether disynaptic motifs^64^ occurred significantly more frequently than expected by chance, we simulated the entire set of recording configurations including PV^+^ interneurons (41 octuples, 62 septuples, 54 sextuples, 37 quintuples, 14 quadruples, 7 triples, and 3 pairs in 218 slices) 10,000 times, assuming random connectivity^14,38,64^. The connection probabilities were set to the experimentally determined distance-dependent values. For each simulated data set, we counted the number of all 25 possible disynaptic motifs (Fig. 5a). From the 10,000 bootstrap replications, mean, median, and confidence intervals for these counts were determined. *P* values were calculated as the number of replications in which the motif number was equal to or larger than the empirical number, divided by the number of replications. If a motif was never encountered in the 10,000 replications, *P* was assumed as < 0.0001. For assessing statistical significance, correction for multiple testing was performed using a Benjamini–Hochberg method that controls the false discovery rate^65^. *P* values for m comparisons were sorted in increasing order (*P*_1_ ≤ *P*_2_ ≤ … ≤ *P*_m_), the first *P*_i_ value that satisfied the condition *P*_i_ ≤ i / m 0.05 was identified (starting with *P*_m_), and the motifs corresponding to *P*_j_ values with 1 ≤ j ≤ i were considered significant. For illustration purposes, *P* values were converted into z scores, using the quantiles of a standard normal distribution.

### Morphological analysis

Neurons that were filled with biocytin (0.3%) for >1 h were processed for morphological analysis. After withdrawal of the pipettes, resulting in the formation of outside-out patches at the pipette tips, slices were fixed for 12–24 h at 4 °C in a 0.1 M phosphate buffer (PB) solution containing 2.5% paraformaldehyde, 1.25% glutaraldehyde, and 15% (v/v) saturated picric acid solution. After fixation, slices were treated with hydrogen peroxide (1%, 10 min) to block endogenous peroxidases, and rinsed in PB several times. Membranes were permeabilized with 1% Triton X100 in PB for 1 h. Slices were then transferred to a PB solution containing 1% avidin-biotinylated horseradish peroxidase complex (ABC, Vector Laboratories) and 1% Triton X100 for ~ 12 h. Excess ABC was removed by several rinses in PB and the slices were developed with 0.05% 3,3′-diaminobenzidine tetrahydrochloride (DAB) and subsequently hydrogen peroxide. Finally, slices were embedded in Mowiol (Sigma-Aldrich).

### In vivo recordings from dentate gyrus PV^+^ interneurons

Whole-cell patch-clamp recordings in vivo were performed in male 35- to 63-day-old mice as described previously^53^. Animals were in the head-fixed, fully awake configuration, and were running on a linear belt treadmill^66,67^. The head-bar implantation and craniotomy were performed under anesthesia by intraperitoneal injection of 80 mg/kg ketamine (Intervet) and 8 mg/kg xylazine (Graeub), followed by local anesthesia with lidocaine. A custom-made steel head-bar was attached to the skull using superglue and dental cement. The day before recording, two small (~0.5 mm in diameter) craniotomies, one for the patch electrode and one for a local field potential (LFP) electrode, were drilled at the following coordinates: 2.0 mm caudal, 1.2 mm lateral for whole-cell recording; 2.5 mm caudal, 1.2 mm lateral for the LFP recording. The dura was left intact, and craniotomies were covered with silicone elastomer (Kwik-Cast, World Precision Instruments). Pipettes were fabricated from borosilicate glass capillaries (1.75 mm outer diameter, 1.25 mm inner diameter). Long-taper whole-cell patch electrodes (9–12 MΩ) were filled with a solution containing: 130 mM K-gluconate, 2 mM KCl, 2 mM MgCl_2_, 2 mM Na_2_ATP, 0.3 mM NaGTP, 10 mM HEPES, 18 mM sucrose, 10 or 0.1 mM EGTA, and 0.3% biocytin, pH adjusted to 7.28 with KOH. Whole-cell patch electrodes were advanced through the cortex with 500–600 mbar of pressure to prevent the electrode tip from clogging. After passing the hippocampus CA1 subfield, the pressure was reduced to 20 mbar. After the blind whole-cell recording was obtained, series resistance was calculated by applying a test pulse ( + 50 mV and −10 mV) under voltage-clamp conditions. Recordings were immediately discarded if series resistance exceeded 100 MΩ. After the bridge balance was compensated, step currents from −100 pA to 400 pA were injected to calculate input resistance and maximal firing frequency of the recorded cells. All the recordings were done in current-clamp experiment configuration without holding current injection using a Heka EPC double amplifier. Signals were low-pass filtered at 10 kHz (Bessel) and sampled at 25 kHz with Heka Patchmaster acquisition software. After recording, the patch pipettes were slowly withdrawn to form an outside-out patch, verifying the integrity of the seal. Data included were obtained from three fast-spiking cells in the dentate gyrus, which generated APs during sustained current injection at a frequency of >100 Hz. To determine the relative AP threshold, spontaneous action potentials (sAPs) were detected, using either a single sAP or the first AP in a burst. The membrane potential preceding the sAP was measured in a 10–20 ms time window before the sAP. sAP absolute threshold was determined from a d*V*/d*t*–*V* phase plot; the rising phase was fit with an exponential function including a shift factor, and the intersection of the fit curve with the baseline was defined as threshold.

## Electronic supplementary material

Supplementary Information

## Data Availability

Original data, analysis programs, and computer code will be provided by the corresponding author (P.J.) upon request.
