# Supplementary material for: Parvalbumin+ interneurons obey unique connectivity rules and establish a powerful lateral-inhibition microcircuit in dentate gyrus
Source: Nat Commun. 2018 Nov 2;9:4605. doi: 10.1038/s41467-018-06899-3 (PMC6214995; doi:10.1038/s41467-018-06899-3)
Supplement: Supplementary file 1 — Supplementary Information [file 41467_2018_6899_MOESM1_ESM.pdf]

Supplementary Figures

Supplementary Figure 1 | Action potential phenotype and membrane properties of different types of genetically identified interneurons in the dentate gyrus.

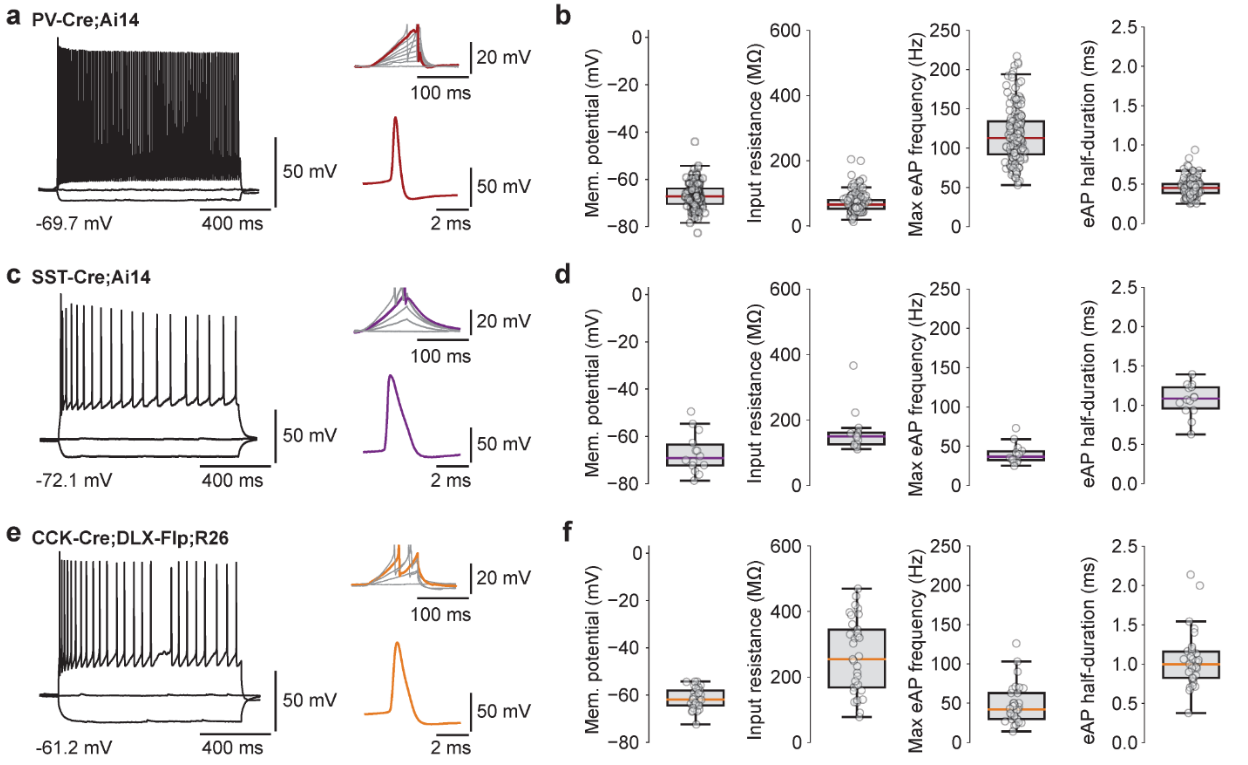

(**a**) Functional properties of identified PV$^+$ interneurons in the dentate gyrus. Left, voltage changes evoked by long depolarizing and hyperpolarizing current pulses (0.6, 0, and −0.1 nA) applied to the PV$^+$ interneuron. Fast-spiking phenotype (> 100 Hz) and low input resistance (< 100 MΩ) are characteristic. Right, single AP waveform evoked by a depolarizing current ramp.

(**b**) Box plots of resting membrane potential (mean, –66.9 mV), input resistance (70.2 MΩ), maximal evoked AP frequency (116 Hz), and evoked AP half-duration (0.45 ms; 173 cells). PV$^+$ interneurons were identified in slices based on tdTomato labeling in PV-Cre;Ai14 mice.

(**c, d**) Similar data as in (a, b), but for SST$^+$ interneurons. SST-Cre mice were used for labeling.

(**e, f**) Similar data as in (a, b), but for CCK^{+} interneurons. CCK-Cre;DLX 5/6-Flp mice were used for labeling. In (c, e), voltage changes were evoked by long depolarizing and hyperpolarizing current pulses (0.3, 0, and −0.1 nA).

Box plots in (b, d, and f) show lower quartile (Q1), median (horizontal red line), and upper quartile (Q3). The interquartile range (IQR = Q3–Q1) is represented as the height of the box. Whiskers extend to the most extreme data point that is no more than 1.5 x IQR from the edge of the box (Tukey style). Data from individual cells are plotted on top of the corresponding box.

Supplementary Figure 2 | Coactivation of converging inputs from granule cells is required to initiate APs in PV$^+$ interneurons.

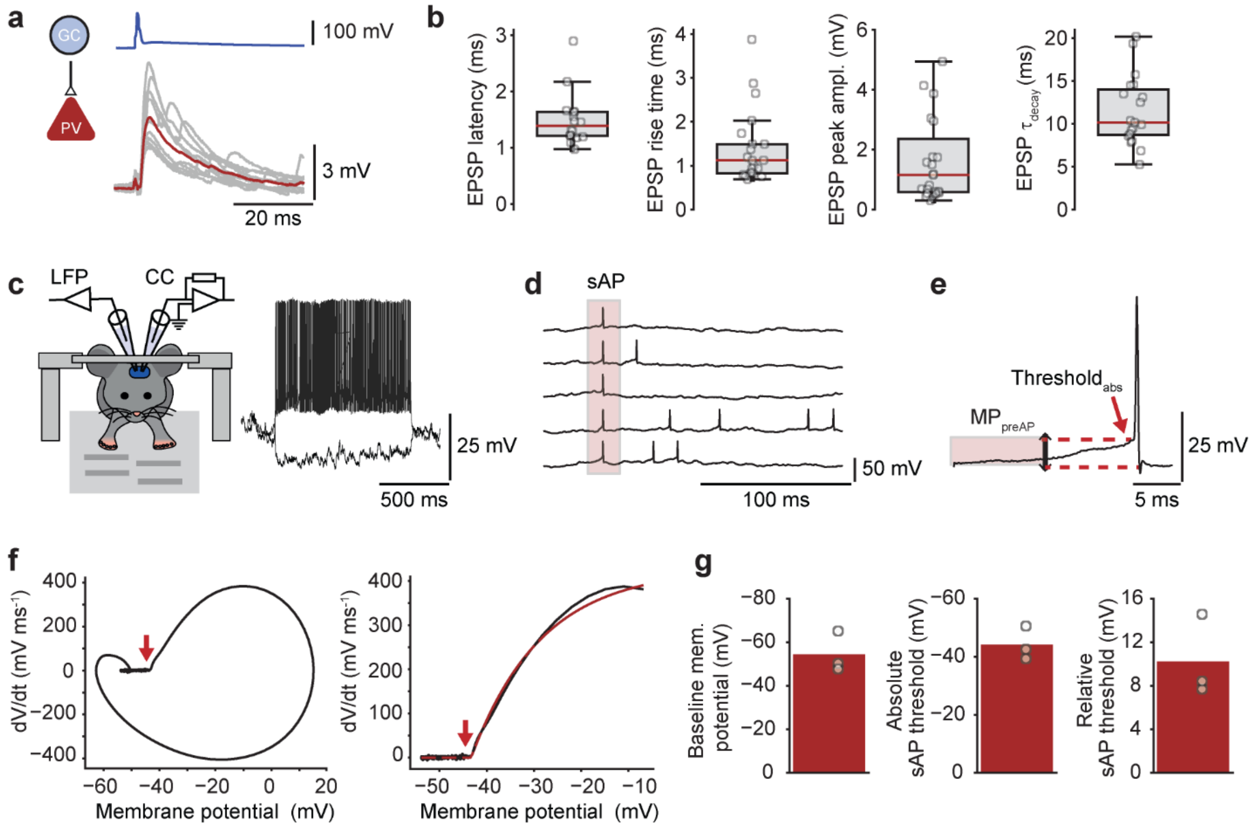

(**a**) Unitary excitatory postsynaptic potentials (EPSPs), with individual synaptic responses (gray) and average trace (red, 15 traces) in a representative GC–PV$^+$ interneuron pair.

(**b**) Box plots of EPSP latency, 20–80% rise time, peak amplitude, and decay time constant.

(**c**) AP properties of fast-spiking interneurons in the dentate gyrus *in vivo* in awake, behaving animals. Left, schematic illustration of recording configuration; CC, whole-cell current clamp; LFP, local field potential recording. Right, traces of membrane potential in response to depolarizing and hyperpolarizing current injections.

(**d, e**) Compressed plot of five spontaneous APs (sAPs) aligned to the sAP peak (d) and expanded plot of a single AP (e). Light red area shows the time interval in which the

baseline membrane potential before the spike was determined. MP_{preAP}, membrane potential preceding AP.

(**f**) Left, phase plot analysis of the AP shown in (e). Right, fit of the rising component of the phase plot by an exponential function including a shift factor (red curve). Red arrows indicate absolute threshold of sAP initiation determined from the shift.

(**g**) Summary bar graph of baseline membrane potential (in time window 10–20 ms before the AP, left), absolute sAP threshold (center), and relative sAP threshold (relative threshold = absolute threshold – baseline membrane potential).

Supplementary Figure 3 | Propagation of evoked EPSPs in PV^{+} interneuron networks via gap junctions.

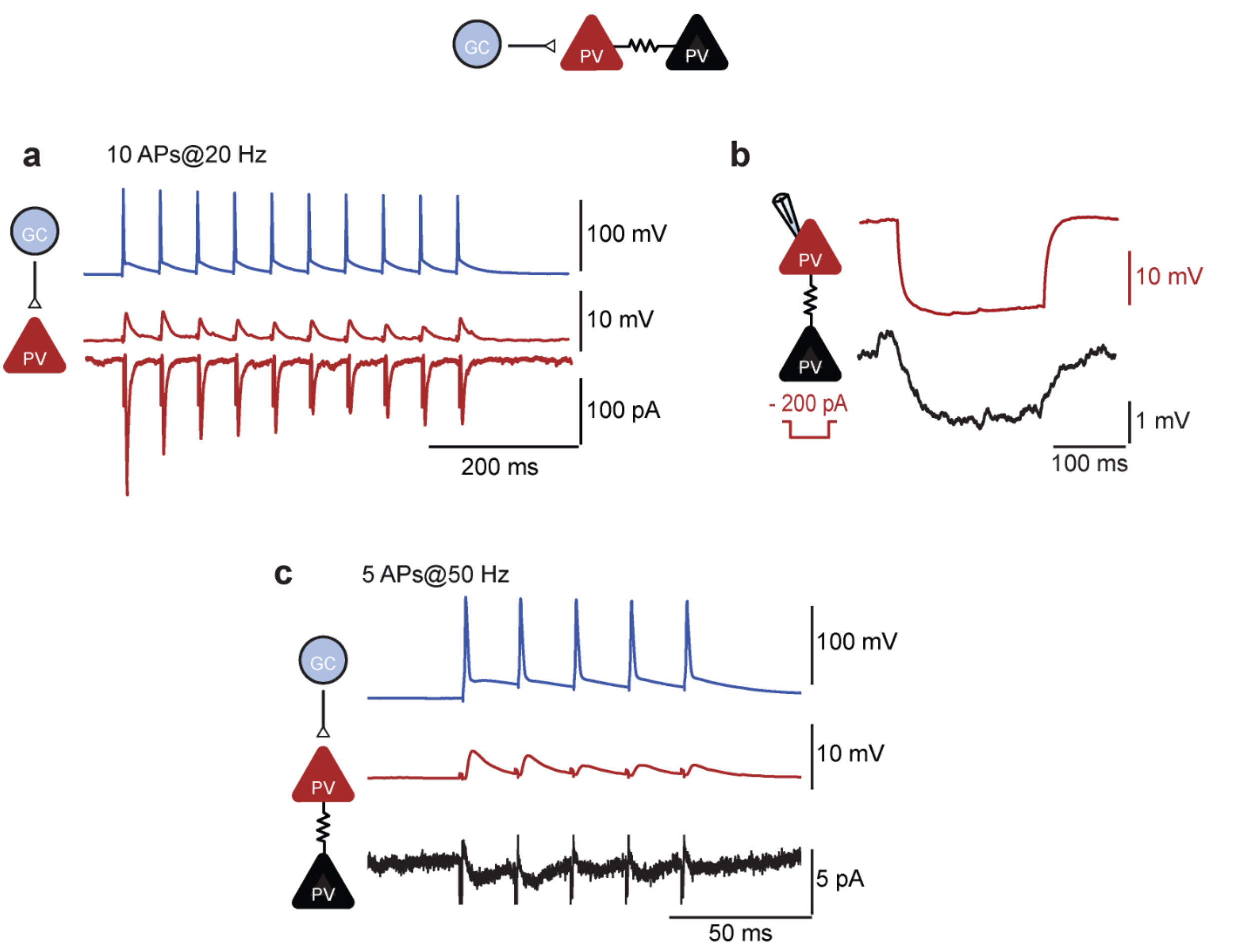

(**a**) Simultaneous recording from a GC and two PV^{+} interneurons. APs in the GC led to monosynaptic EPSPs (current-clamp conditions) or EPSCs (voltage-clamp conditions) in the first PV^{+} interneuron.

(**b**) The two recorded PV^{+} interneurons were coupled by gap junctions.

(**c**) Propagation of EPSPs from PV^{+} interneuron 1 (red) to PV^{+} interneuron 2 (black). APs in the GC led to EPSCs with peak amplitude < 5 pA in the second PV^{+} interneuron. These excitatory synaptic events were apparently propagated via gap junctions[S1].

Supplementary Figure 4 | Relation between intersomatic distance and axon length.

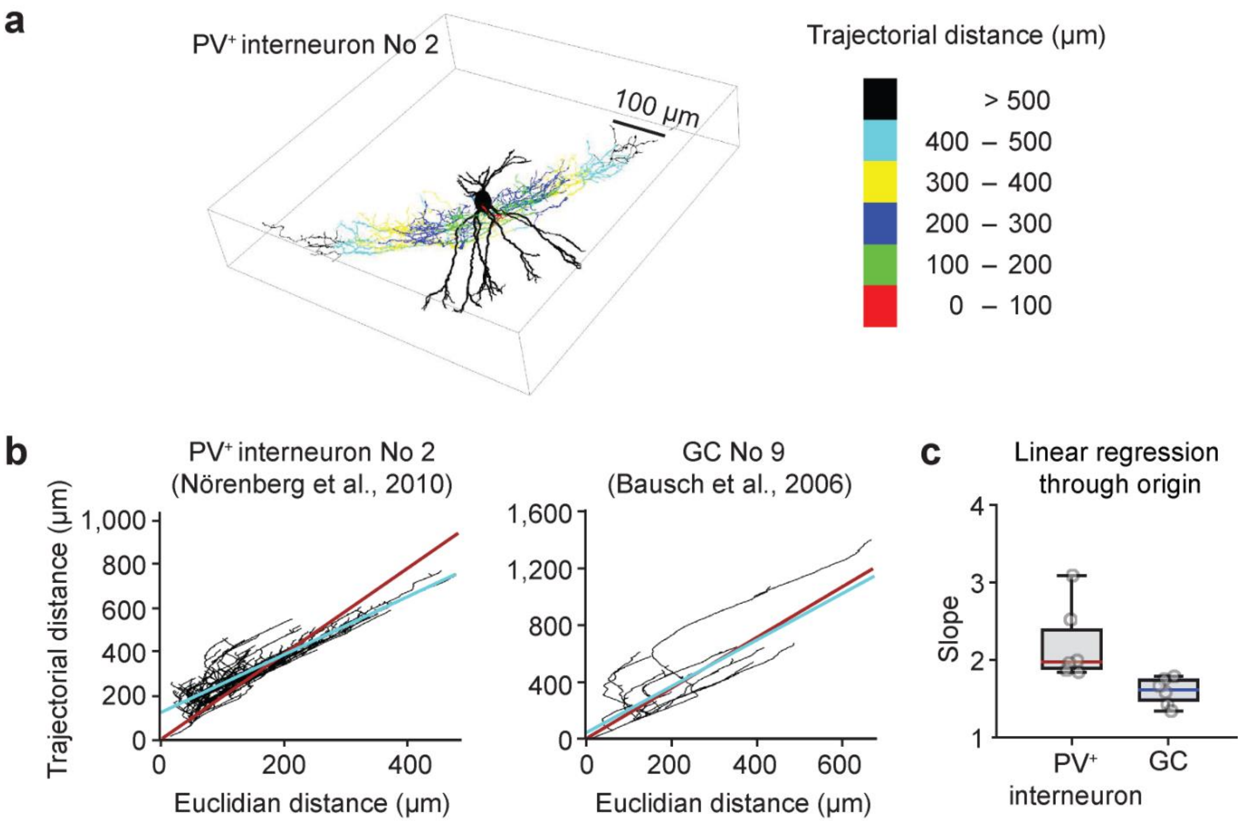

(**a**) Reconstructed fast-spiking PV$^+$ interneuron in the dentate gyrus. Soma and dendrites are drawn in black, axon is color coded according to the length of the axonal path. Cell No 2 from the sample of dentate gyrus PV$^+$ interneurons from Nörenberg et al.[S2].

(**b**) Plot of axon length (trajectorial distance) against intersomatic distance (Euclidian distance). Data points were analyzed by regression with linear function through origin (red line) or with offset (light blue line). Left, PV$^+$ interneuron (cell No 2; Ref. S2); right, GC (cell No 9; Ref. S3). All distance values were measured relative to the center of the soma.

(**c**) Slope of the axon length–intersomatic distance relation (fit with line through origin). Box plots show lower quartile (Q1), median (horizontal red line), and upper quartile (Q3). The interquartile range (IQR = Q3–Q1) is represented as the height of the box. Whiskers extend to the most extreme data point that is no more than 1.5 x IQR from the edge of the box (Tukey style). Data from individual cells are plotted on top of the corresponding

box. Data from six fast-spiking PV^+ interneurons in the dentate gyrus and six dentate gyrus GCs (Cells No 9, 47, 52, 56, 58, and 61; Ref. S3).

Supplementary Table 1 | Abundance of lateral inhibition in different brain regions.

Brain region	p_{IE} / p_{EI} [a]	$n_{lateral}$ / $n_{recurrent}$ [b]	Reference
Visual and somatosensory cortex	0.99	0.13	Holmgren et al., 2003 [S4]
Visual cortex	2.5	2.0	Yoshimura and Callaway, 2005 [S5]
Entorhinal cortex	1.5	1.1	Couey et al., 2013 [S6]
Presubiculum superficial	1.04	1.3	Peng et al., 2017 [S7]
Presubiculum deep	0.68	0.67	Peng et al., 2017 [S7]
Dentate gyrus	3.83 [c]	9.25	This paper

(a) p_{IE} / p_{EI} indicates ratio of mean inhibitory IN–PN to mean excitatory PN–IN connection probability.

(b) $n_{lateral}$ / $n_{recurrent}$ represents ratio of number of lateral inhibition motifs and recurrent inhibition motifs in all recorded PN–IN pairs.

(c) Quantified from the integral under the connection probability–distance curves (**Fig. 3**).

Supplementary References

S1. Galarreta, M. & Hestrin, S. A network of fast-spiking cells in the neocortex connected by electrical synapses. *Nature* **402**, 72–75 (1999).

S2. Nörenberg, A., Hu, H., Vida, I., Bartos, M. & Jonas, P. Distinct nonuniform cable properties optimize rapid and efficient activation of fast-spiking GABAergic interneurons. *Proc. Natl. Acad. Sci. USA* **107**, 894–899 (2010).

S3. Bausch, S.B., He, S. & Dong, Y. Inverse relationship between seizure expression and extrasynaptic NMDAR function following chronic NMDAR inhibition. *Epilepsia* **51** Suppl 3, 102–105 (2010).

S4. Holmgren, C., Harkany, T., Svennenfors, B. & Zilberter, Y. Pyramidal cell communication within local networks in layer 2/3 of rat neocortex. *J. Physiol.* **551**, 139–153 (2003).

S5. Yoshimura, Y. & Callaway, E.M. Fine-scale specificity of cortical networks depends on inhibitory cell type and connectivity. *Nat. Neurosci.* **8**, 1552–1559 (2005).

S6. Couey, J.J., Witoelar, A., Zhang, S.J., Zheng, K., Ye, J., Dunn, B., Czajkowski, R., Moser, M.B., Moser, E.I., Roudi, Y. & Witter, M.P. Recurrent inhibitory circuitry as a mechanism for grid formation. *Nat. Neurosci.* **16**, 318–324 (2013).

S7. Peng, Y., Barreda Tomás, F.J., Klisch, C., Vida, I. & Geiger, J.R.P. Layer-specific organization of local excitatory and inhibitory synaptic connectivity in the rat presubiculum. *Cereb. Cortex* **27**, 2435–2452 (2017).
